# Supplementary material for: Differential stimulation of the retina with subretinally injected exogenous neurotransmitter: A biomimetic alternative to electrical stimulation
Source: Sci Rep. 2016 Dec 8;6:38505. doi: 10.1038/srep38505 (PMC5144088; doi:10.1038/srep38505)
Supplement: Supplementary Information [file srep38505-s1.pdf]

Supplementary Material

Differential stimulation of the retina with subretinally injected exogenous neurotransmitter: A biomimetic alternative to electrical stimulation

Corey M. Rountree[1,†,§], Samsoon Inayat[2,‡,§], John B. Troy[1], Laxman Saggere[2*]

[1]Department of Biomedical Engineering, Northwestern University, Evanston, Illinois 60208, USA.

[2]Department of Mechanical and Industrial Engineering, University of Illinois at Chicago, Chicago, Illinois 60607, USA.

[†]Present Address: Department of Mechanical and Industrial Engineering, University of Illinois at Chicago, Chicago, Illinois 60607, USA.

[‡]Present Address: Department of Neuroscience, University of Lethbridge, Lethbridge, Alberta T1K 3M4, Canada.

[§]These authors contributed equally to this work.

[*]Correspondence and requests for materials should be addressed to L.S. (email: saggere@uic.edu).

This PDF file includes:

Supplementary Figures: S1 to S7

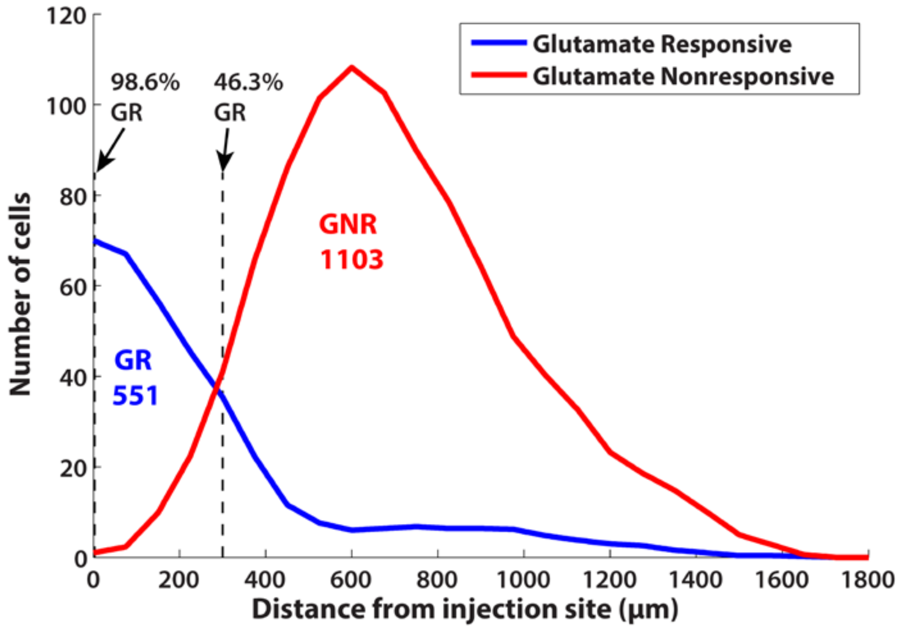

Supplementary Figure S1. Spatial distributions of responsive and non-responsive cells relative to the glutamate injection site. The plot shows how the total numbers of glutamate-responsive (GR) and glutamate-nonresponsive (GNR) units across all retina samples varied with distance relative to the injection site. GR units (blue) were predominately confined near the injection site and decreased with increasing distances while GNR units (red) were rare near the site of injection and were most prominent at distances around 600 μm. The relative percentages of GR cells at two distances (0 and 300 μm) are shown with black dashed lines to highlight the interplay between the GR and GNR distributions. As can be seen, approximately 99% of cells (70 of 71) were GR at the injection site, but only 46% of cells responded to glutamate injections at 300 μm distance from the injection site. The right edges of both GR and GNR distributions in the plot were forced to zero due to the limited sampling area of our multielectrode array (MEA) where the farthest electrodes were separated by a distance of ~1700 μm.

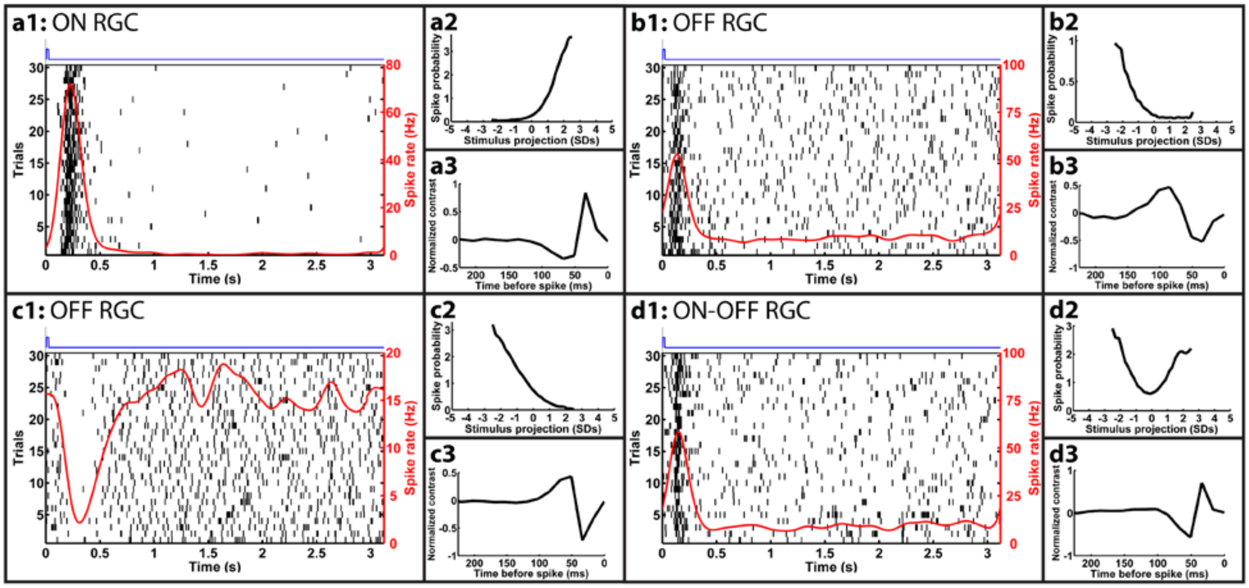

Supplementary Figure S2. Subretinal glutamate injections elicit both excitatory and inhibitory responses from RGC subtypes. (**a**) Plot a1 shows the raster and spike rate plot for an ON RGC to a 20 ms injection of glutamate (indicated by blue trace at top) below the photoreceptor surface, which produced a robust, excitatory response. Each vertical black line on this plot represents a single spike and trials are stacked vertically (left y-axis). The red line (right y-axis) shows the spike rate through time. Plot a2 is the static nonlinearity calculated through STC-NC analysis, which indicates the probability of this cell responding to light (stimulus projections greater than 0) or dark (stimulus projections less than 0). This cell only has high spike probabilities when presented with light stimuli, demonstrating that it is an ON-center RGC. Plot a3 is a representation of this ON RGC's STA timecourse, which tracks the average contrast of the STA in the receptive field during the interval preceding a spike. (**b**), (**c**) and (**d**) Similar results are shown for an OFF RGC with an excitatory response to glutamate (b), an OFF RGC with an inhibitory glutamate response (c), and an excitatory ON-OFF RGC (d).

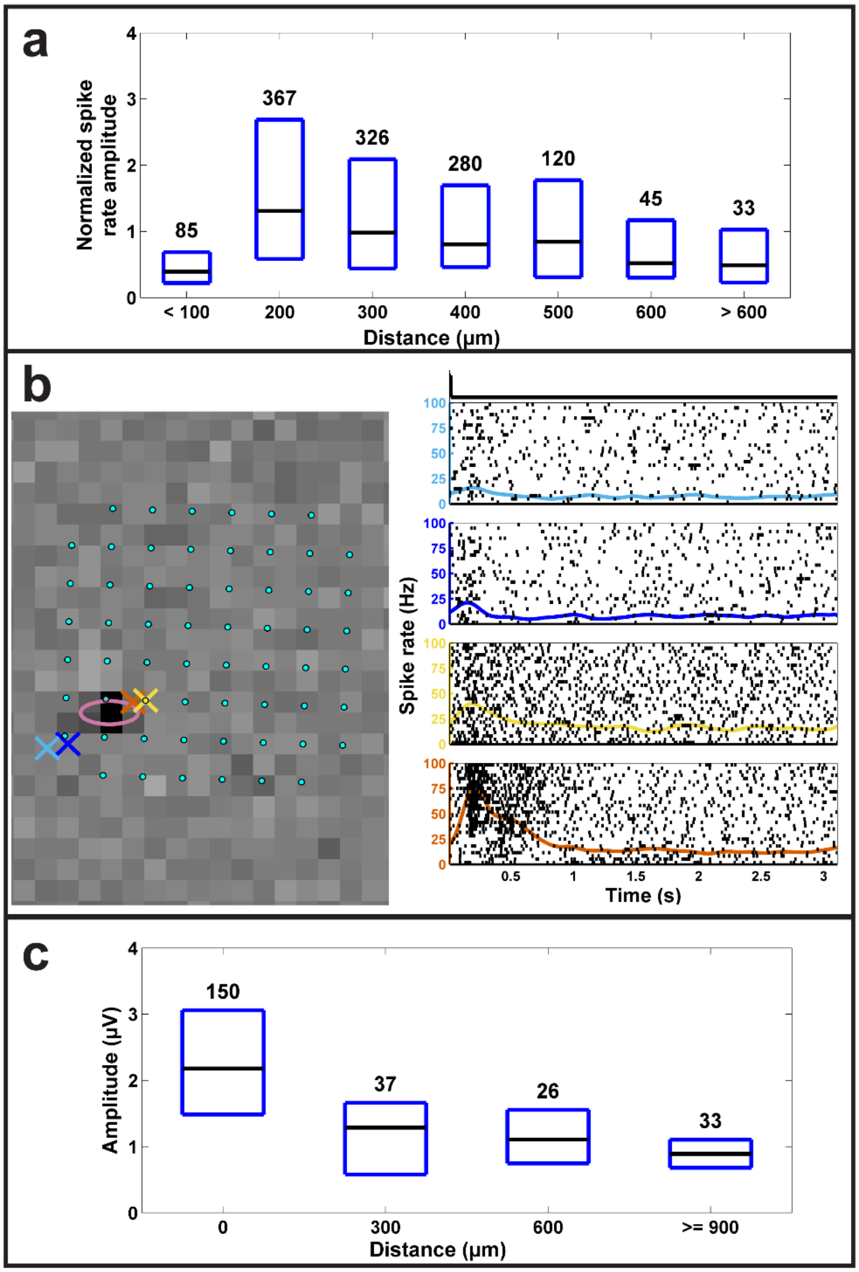

Supplementary Figure S3. RGC response amplitudes decrease with increasing distances of the RF centers of RGCs from the injection site. (**a**) A comparison of the normalized amplitude of responses at different distances from the RF center. The amplitudes are normalized by dividing the glutamate amplitudes by light-evoked amplitudes to allow comparison of responses with wide variations in basal spike rates.

Injections near (< 200 μm) the RF center elicited amplitudes comparable to light responses and were significantly ($p < 0.001$, Kruskal Wallis) larger than those farther away. Boxes show median, lower and upper quartiles while the numbers above boxes indicate sample sizes. (**b**) A map (left side) showing the locations of glutamate injections (colored '×'s) with respect to the RF of an OFF RGC (magenta ellipse) superimposed over this cell's peak STA frame. The cyan circles represent the locations (200 μm apart) of the 60 electrodes of the MEA. The spike rate and raster plots on the right side show the responses to several identical glutamate injections corresponding to the '×'s in the spatial map arranged from farthest (top) to closest (bottom). (**c**) A comparison of gLFP amplitudes, showing that gLFP amplitude decreases with distance from the injection site. The gLFP amplitudes near the injection site (≤ 300 μm) were significantly larger ($p < 0.05$, Kruskal Wallis) than those farther away.

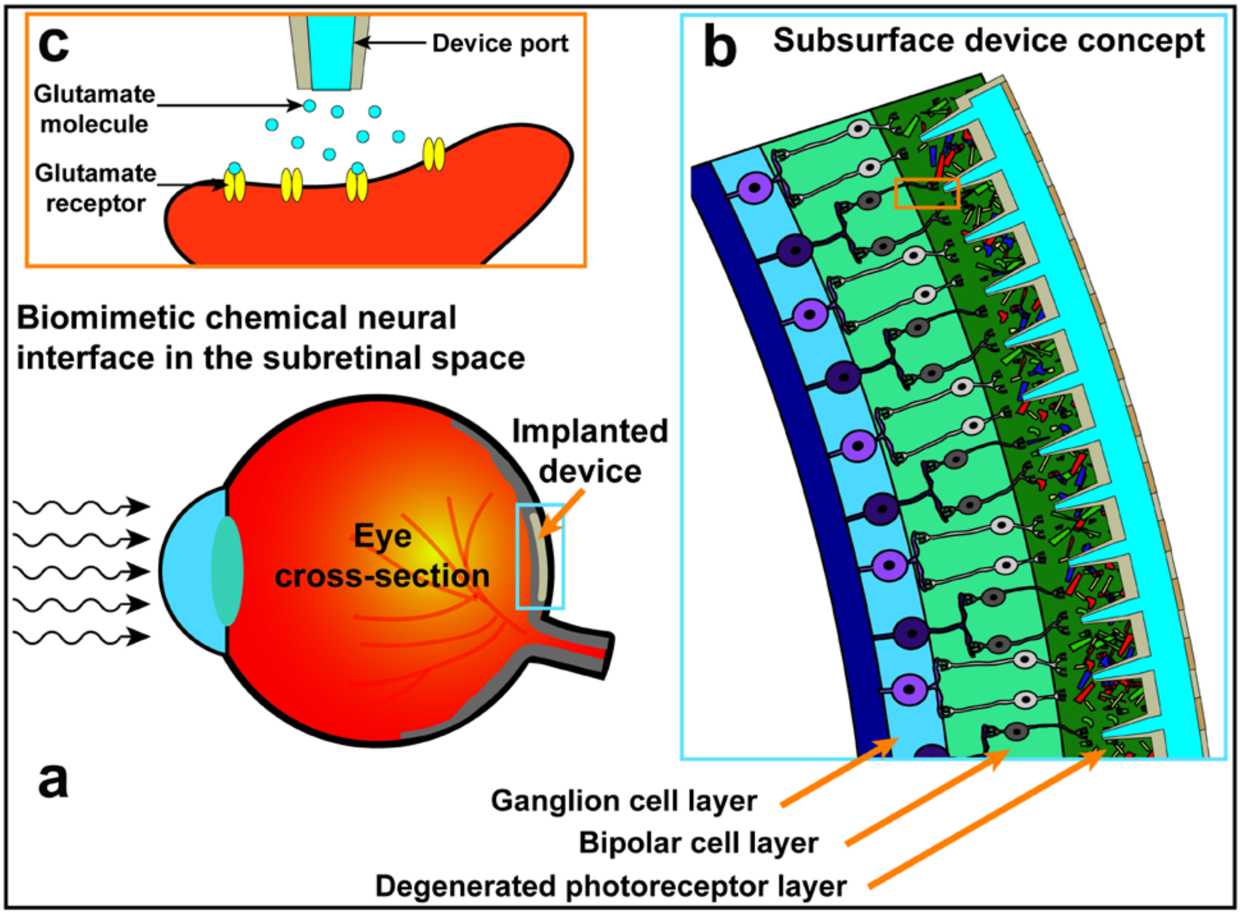

Supplementary Figure S4. Concept of a neurotransmitter-based subretinal prosthesis. (**a**) A chemical-based prosthesis implanted in the eye. (**b**) Close-up detail of the prosthesis interfaced with the retina in the subretinal space of a PR degenerated retina. Such a device, enabled by MEMS technology, would comprise on-chip reservoirs storing a neurotransmitter chemical, a high density array of independently addressable microneedles and tiny light-powered actuators (not shown in the illustration). (**c**) When activated, the actuators would eject small, pulsatile boluses of the stored chemical on to target INL synapses through the microneedle ports. Thus, the device interacts with glutamate receptors of surviving cells, and, in principle, mimics the functionality of a normal photoreceptor layer.

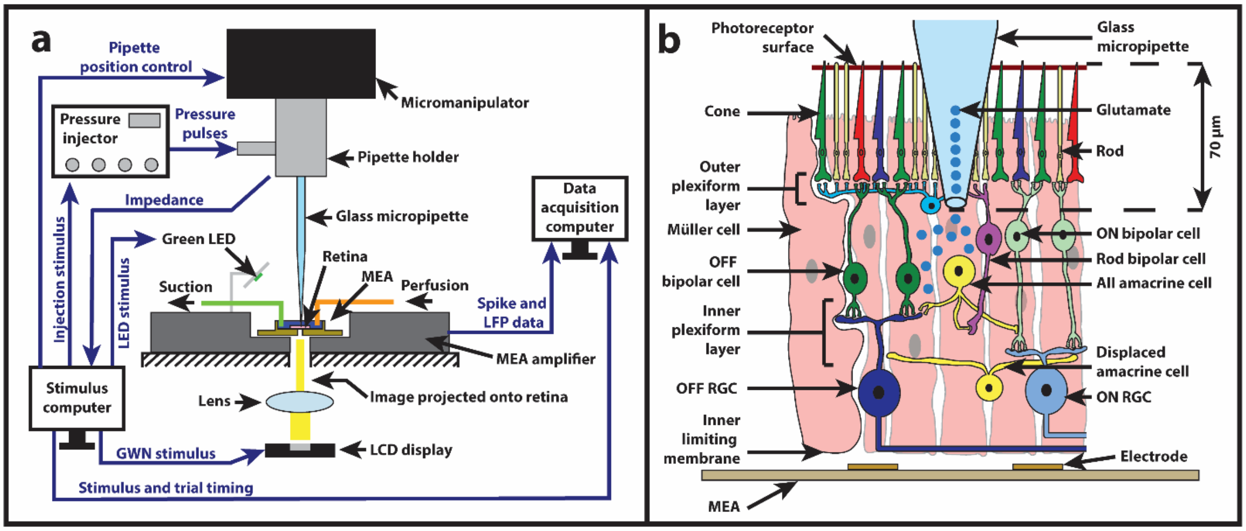

Supplementary Figure S5. Experimental setup and close-up details of the glutamate injections. (**a**) Schematic of the experimental platform showing the arrangement of various components. Retinas isolated from rat eyes were placed on a multielectrode array (MEA) with retinal ganglion cell (RGC) side towards the electrodes to record local field potentials (LFPs) and RGC spiking activity using a data acquisition computer. Visual stimulations were accomplished using a small SVGA monitor and a green LED based on signals from a dedicated computer monitoring the stimuli. Glutamate was injected into the retina from the photoreceptor side via a micropipette using a pressure injection system (also controlled by the stimulus computer). The flow of output signals and input data throughout the setup is represented by the blue lines with labels above. (**b**) A close-up view of the micropipette and the retina interface in (a), depicting the location of subretinal glutamate injections, approximately 70 µm inside the retina near the synapses of the OPL, with respect to the retinal anatomy.

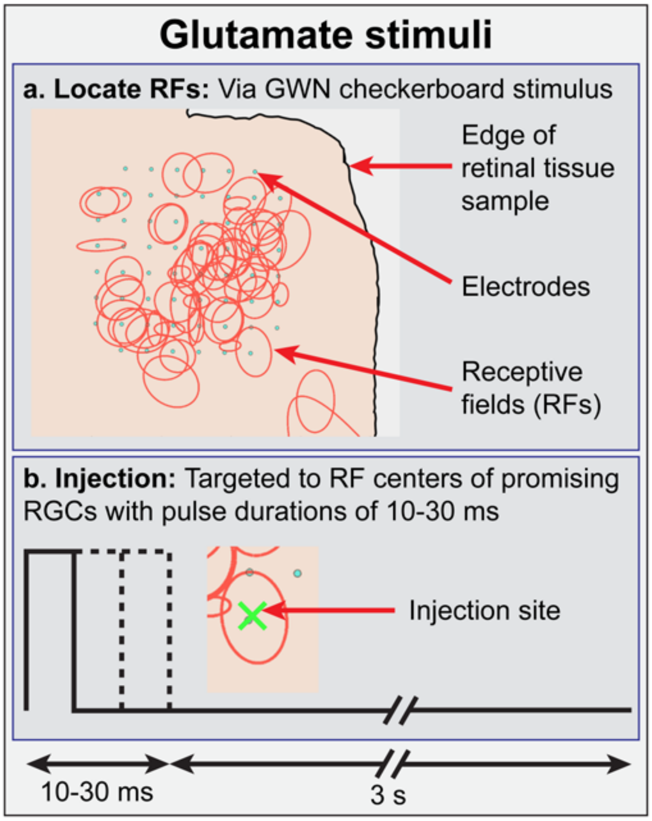

Supplementary Figure S6. Overview of glutamate stimulation methodology. Glutamate stimulation trials proceeded in two major steps. (**a**) First, RGC receptive fields were located using GWN checkerboard stimulation followed by STA and STC-NC analysis. (**b**) Second, a glutamate-filled pipette was positioned at the RF center of a visually-responsive cell and inserted 70 μm subsurface before using pressure pulses of durations 10-30 ms (represented by black lines) to release glutamate near the target cell. Most glutamate stimulation trials were conducted at a constant interpulse duration of 3 s. In some studies however, the interpulse duration was varied between 150 to 4000 ms while maintaining a constant pulse width of 10 ms to study the temporal resolution of glutamate stimulation at injection frequencies ranging between 0.25-6 Hz.

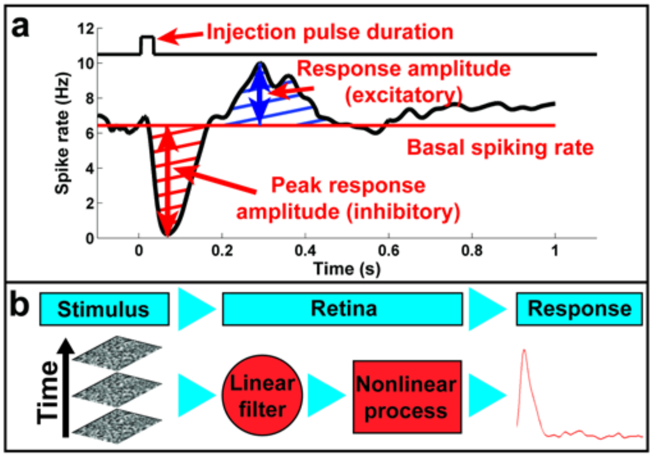

Supplementary Figure S7. Spike rate and GWN stimulation analyses. (**a**) Definition of spike rate amplitude and excitatory/inhibitory phases in a representative spike rate curve. Since the peak response is inhibitory, this is considered an inhibitory response. (**b**) Diagram of the process of spike triggered covariance non-centered (STC-NC) analysis. Spike responses to Gaussian white noise checkerboard stimulation are used to estimate a linear filter and nonlinear process for each cell. The linear filter is calculated as the spike-triggered average (STA) while the STC-NC estimates the nonlinear component, which can be further used to classify subtypes. This nonlinearity represents the probability of spike generation in response to different contrasts of visual stimuli, where positive and negative nonlinearities represent biases towards light (ON cell) and dark (OFF cell) stimuli, respectively. Biases near zero indicate cells with both light and dark responses which identifies them as ON-OFF RGCs.
